# Outcome and prognostic markers of cirrhotic and non-cirrhotic patients admitted to a hepatology ICU in a tertiary care university hospital

**DOI:** 10.4314/ahs.v22i2.43

**Published:** 2022-06

**Authors:** Zeinab Abdellatif, Rasha Eletreby, Reham Samir, Mohamed Abdallah, Rania Lithy, Naglaa Zayed

**Affiliations:** 1 Endemic Medicine and Hepatology Department, Faculty of Medicine, Cairo University, Cairo, Egypt; 2 National Research Institute, Cairo, Egypt

**Keywords:** Liver cirrhosis, ICU, SOFA score, mortality

## Abstract

**Background and aim:**

Patients with decompensated liver cirrhosis are not given priority for ICU admission in the settings of limited place availability. Recently, advances in medical care led to improvement in their survival. Our aim is to study the outcome of patients admitted to our hepatology ICU.

**Methods:**

We retrieved the data of patients admitted to the Endemic Medicine Department ICU at Kasr Al-Ainy Hospital in the period from November 2014 to May 2018. We included 498 patients who had complete clinical and outcome data in this analysis. The primary outcome was ICU mortality and its predictors.

**Results:**

The overall mortality was 48.1% in the liver cirrhosis versus 52.9% in the non-cirrhosis group. The most common presentations of cirrhotic patients were hepatic encephalopathy and hypovolemic shock. The SOFA score and sepsis independently predicted mortality in the overall cohort.

**Conclusion:**

The mortality of cirrhotic patients admitted to ICU is not higher than non-cirrhotic patients. SOFA score is a good prognostic indicator in patients with cirrhosis.

## Introduction

Decompensated liver cirrhosis is associated with high rates of hospitalization, increased length of stay, cost, mortality and early readmissions 1, 2. The most common presentations at ICU admission are varicealbleeding, hepatic encephalopathy, acute kidney injury, and patients are usually at increased risk of infection, which predisposes them to the development of organ failure, increased cost of care, and worse outcome^3^.

It was reported that the mortality rate of patients with cirrhosis admitted to an intensive care unit (ICU) due to organ dysfunction ranges from 34% to 69% depending on the cause of admission, the presence of organ failure (OF) and the severity of the underlying liver disease. ICU mortality has markedly decreased over the last years from around 90–100% to 41–50% in some reports 3, 4.

Several prognostic models have been utilized in the ICU setting, evaluating severity of illness and quantifying organ dysfunction and failure. Some are ICU-specific scores, such as the Sequential Organ Failure Assessment (SOFA) score 5, the Acute Physiology and Chronic Health Evaluation (APACHE)^6^, and others are disease-specific models as Child-Pugh (CTP) score 7, the standard Model for End-Stage Liver Disease (MELD) score 8 and MELD-serum sodium score.

The current study aims to assess mainly the outcome of critically ill cirrhotic and non-cirrhotic patients admitted to a hepatology ICU at a tertiary level teaching hospital.

A second aim is to explore potential predictors of ICU mortality that may allow risk stratification at the time of ICU admission.

## Patients and methods

### Study design

This retrospective observational study included patients who were admitted to a hepto- gastroenterology intensive care unit, at the Endemic Medicine Department, KasrAlainy Hospital, Faculty of Medicine, Cairo University from November 2015 to May 2018.

### Ethics

The study was reviewed and approved by the Institutional Review Board (IRB) of Faculty of Medicine, Cairo University and in compliance with the ethics principles of the 1975 Declaration of Helsinki.

### Variables and outcome measures

At ICU admission, several variables were collected for each patient:

Demographic variables included age, gender, presence or absence of co morbidities.

The cause of ICU admission according to the physician primary diagnosis was described e.g. Disturbed conscious level, GIT bleeding, SBP, respiratory failure, renal impairment, sepsis.

Events during ICU stay; GI bleeding, organ failure, development of aspiration pneumonia.

Duration of ICU stay

Relevant liver-specific prognostic models [CTP, as well as general ICU models (APACHE II and SOFA scores) were evaluated on ICU admission.

Prognostic markers as well as clinical outcome were collected for each patient.

Liver cirrhosis was diagnosed on clinical basis together with its characteristic ultrasonographic features, laboratory tests and/or liver stiffness measurement by transient elastography of ≥12.5 kPa^9^. The most common underlying cause of liver cirrhosis was chronic HCV infection.

### Outcome measures

ICU mortality and possible factors predicting mortality. Possible association between these variables and prognostic models that can predict ICU mortality were analyzed.

### Statistical analysis

Descriptive statistics were done. Baseline demographic, clinical and prognostic scores parameters were compared between the 2 groups, using the Chi square test for categorical variables and the Wilcoxon rank sum test for non-normally distributed numerical variables.

Logistic regression analysis was done to identify possible predictors of mortality in each group. Multivariable regression model was done to evaluate the independent prognostic predictors. Related variables were not included in the same model to avoid collinearity. All tests were two-sided and p<0.05 are deemed significant. Statistical analysis of the data was performed using STATA 13.1 statistical software (Copyright 1985–2013 StataCorp LP, College Station, Texas 77845 USA).

## Results

### 1. Baseline patients' characteristics

Seven hundred and fifty patients were admitted to the hepto- gastroenterology intensive care unit, at the Endemic Medicine Department, KasrAlainy Hospital, Faculty of Medicine, Cairo University from November 2015 to May 2018. The analysis was performed on 498 patients whose relevant clinical and outcome data were recorded. Nearly 75.5% of the patients were admitted due to a hepatic cause.

The overall mortality was 50.8% in this cohort, with no significant difference in mortality between patients with liver cirrhosis and those without, table 1.

**Table 1:** Outcome of the critically ill patients admitted to the ICU by the presence of liver cirrhosis

	Liver Cirrhosis (n=322)	No liver cirrhosis (n=170)	*P* value
**Improved and discharged** **alive**	159 (49.4%)	76 (44.7%)	
**Died**	**155 (48.1%)**	**90 (52.9%)**	0.6
**Discharged against** **medical advice**	8 (2.5%)	4 (2.35%)	

### 2. Demographic and clinical characteristics of cirrhotic versus non-cirrhotic patients

Liver cirrhosis was present in 61.7% of patients. The median Child-Pugh score was 9 8–10. The median age was higher in the liver cirrhosis group, with male predominance, and a non-statistically significant higher SOFA score, table 2.

**Table 2:** Demographic and clinical characteristics of patients admitted to the ICU categorized by the presence of liver cirrhosis

	Liver Cirrhosis (n=348)		Non-liver cirrhosis (n=186)		*P* value
Age	60 (54–65)		55 (39–65)		0.0001
**Gender** Male/Female	198/150		96/89		0.3
**Duration of** **ICU** **stay (Days)**	4 (3–6)		4 (2–6)		0.8
**DM**	79 (22.7%)		28 (15.1%)		0.04
**Hypertension**	44 (12.6%)		19 (10.2%)		0.4
**SOFA score**	8 (5–11)		7 (4–10)		0.07
**Clinical** **presentation**	Hepatic encephalopathy	115 (33.04%)	Complicated biliary obstruction	49 (26.34%)	
	Hypovolemic shock	82 (23.6%)	Hematemesis (PUD)	15 (8.06%)	
	Sepsis	82 (23.6%)	Endoscopic complications	13 (6.99%)	
	Hematemesis	62 (17.8%)	Acute liver cell failure	6 (3.23%)	
	Complicated biliary obstruction	19	Neutropenic fever	4	
	Endoscopic complications	6	Miscellaneous other causes		
**Status on** **admission**					
DCL	192 (55.2%)		33 (17.74%)		<0.0001
Sepsis	82 (23.6%)		55 (29.6%)		0.1
Hypovolemic shock	82 (23.6%)		41 (22.04%)		0.7
Renal impairment	64 (18.4%)		35 (18.8%)		0.9
Respiratory distress	22 (6.3%)		31 (16.7%)		<0.0001

In the liver cirrhosis group, more than 50% of patients with cirrhosis presented with disturbed conscious level versus 17.7% in the non-cirrhosis group. Sepsis was present in nearly one quarter of patients on admission. The most common cause of admission to ICU was hepatic encephalopathy in this group. Regarding the source of infection, the most common cause was chest infection in around 20% of patients, followed by abdominal infection; namely SBP in 14% and biliary infection in 5.2% of patients.

Patients without background cirrhosis presented mostly with complicated biliary obstruction followed by hematemesis and endoscopic complications.

### 3. Predictors of ICU mortality

Univariable analysis to identify factors associated with the overall mortality is presented in table 3. Themedian age was higher in the non-survivor group. The median SOFA score was significantly higher in the non-survivors, p<0.001. Median Acute Physiologic Assessment and Chronic Health Evaluation (APACHE) II score is also higher in the non-survivors with borderline significance, p=0.05. On multivariate analysis, higher SOFA score and sepsis were predictors of ICU mortality, table 4.

**Table 3:** Univariable analysis of predictors of ICU mortality

	Survivors (n=245)	Non-survivors (n=253)	*P*
**Age**	57 (49–63)	60 (52–66)	0.002
**Gender**			
Male/Female	115/129	146/107	0.3
**Duration of ICU** **stay** (Days)	4 (3–6)	4 (2–6)	0.9
**Diabetes mellitus**	49	51	0.98
**Hypertension**	27	32	0.58
**Hepatic/non-hepatic**	159/76	155/90	0.3
**Cause of ICU** **admission**			
DCL	99	109	0.5
Sepsis	47	80	**0.001**
Hypovolemic shock	55	55	0.8
Renal impairment	24	57	**<0.001**
Respiratory distress	13	37	**0.001**
**Child Pugh** **score** (in cirrhosis)	8 (8–9)	9 (8–10)	0.06
**SOFA score**	6 (4–9)	9 (7–12)	**<0.001**
**APACHE**	14.59 (7.67)	19.33 (7.56)	0.05

SOFA: Sequential Organ Failure Assessment, APACHE: Acute Physiologic Assessment and Chronic Health Evaluation

**Table 4:** Multivariable analysis of predictors of ICU mortality

	OR (95% CI)	*p*
**Age**	1.004 (0.98–1.03)	**0.7**
**SOFA score**	**1.28 (1.14–1.43)**	**<0.001**
Sepsis	**2.28 (1.005–5.19)**	**0.04**

In table 5, predictors of mortality were stratified by the presence of cirrhosis.

**Table 5:** Predictors of ICU mortality by the presence or absence of liver cirrhosis

	Liver cirrhosis			None-cirrhotic		
	Survivors	Non-survivors	*P*	Survivors	Non-survivors	*P*
**Age** Median (IQR)	60 (53–64)	60 (55–66)	**0.02**	48 (36–62)	59.5 (45–66)	**0.02**
**Gender** Male/Female	84/75	92/63	0.2	40/35	50/40	0.8
**DM**	41	33	0.3	8	18	0.09
**Hypertension**	20	21	0.8	7	11	0.5
**DCL**	87	88	0.7	7	19	**0.04**
**Respiratory** **distress**	6	15	**0.04**	7	21	**0.02**
**Renal** **impairment**	17	32	**0.02**	7	24	**0.004**
**Sepsis**	33	38	0.4	14	37	**0.002**
**Presence of** **HCC**	25	39	**0.04**	-		
**SOFA score**	6 (4–9)	10 (7–12)	**<0.0001**	5.5 (2–8)	9 (7–11)	**0.002**
**Child-Pugh** **score**	8 (8–9)	9 (8–10)	**0.06**	-		
**APACHE II** Mean (SD)	15.77 (8.28)	20 (6.81)	0.2	10.75 (3.77)	19.53 (8.06)	0.05

In the liver cirrhosis group, the reported ICU mortality was 48% and SOFA score was the independent predictor of mortality in the liver cirrhosis patients, table 6.

**Table 6:** Multivariable analysis of predictors of ICU mortality

	Liver cirrhosis			None-cirrhotic	
	OR (95% CI)	*p*		OR (95% CI)	*p*
**Age**	1.02 (0.97–1.07)	0.4	**Age**	1.001 (0.97–1.03)	0.97
**HCC**	0.58 (0.17–1.94)	0.4	**Sepsis**	4.52 (1.31–15.53)	0.02
**SOFA** **score**	1.29 (1.11–1.50)	0.001	**SOFA** **score**	1.24 (1.06–1.45)	0.01

In the non-cirrhotic group, the ICU mortality was 53% in this group. Higher SOFA score and the presence of sepsis predicted mortality in this group, table 6.

## Discussion

In the current study, we assessed the outcome of critically ill cirrhotic and non-cirrhotic patients admitted to hepatology ICU at a tertiary level teaching hospital and explored potential predictors of ICU mortality that may allow risk stratification at the time of ICU admission.

Our results revealed that the overall mortality was 50.8%. Sepsis, respiratory distress, renal impairment were the main predictors of mortality by univariate analysis, while only sepsis and high SOFA score on admission remained significant on multivariate analysis.

Mortality rate within cirrhotic patients was 48% which correlates with the reported mortality in the literature of around 41–50%. 3, 4.

Unsurprisingly, most of patients with liver cirrhosis presented with complications of hepatic decompensation necessitating ICU admission. Most commonly, patients presented with advanced hepatic encephalopathy (grade III to IV) (33.04 %). Admission to the ICU is usually indicated for patients with grade III to IV HE who need urgent intervention for airways protection and ventilation. However, grades I and II HE are common in critically ill patients with cirrhosis and other complications such as sepsis, HRS and bleeding. Severe HE necessitating an ICU admission carries a 35% in-hospital & 54% 1-year mortality 10, 11.

Sepsis was observed in 23.5% of the cirrhotic cohort. Chest infection was the most common cause; in nearly 20% of them. According to the literature, the prevalence of infections in cirrhotic patients admitted to the ICU is as high as 59% 12, 13. In the ICU setting, pneumonia is the most common infections seen in cirrhotic patients 12. Causative organisms could be isolated in 50–70% of those patients. In community acquired infections, Gram-negative bacilli (GNB) and Gram-positive cocci (GPC) are the cause in 60% & 30–35% of cases respectively. GPC is seen in 60% of nosocomial infections and usually associated with invasive clinical procedures^12^, ^14^.

Our results revealed that despite being a predictor of mortality in the total cohort; the presence of sepsis didn't predict increased mortality in the cirrhotic patients in particular. This finding agrees with another study which revealed substantial improvement of survival of patients with cirrhosis and septic shock in the recent years; ICU survival is 40% in 2005–2010 vs 17% in 1997–2004. This was attributed to the advances in the management of septic shock and cirrhosis 15. It had been reported that compliance with the international guidelines is associated with improved survival 16. On the other hand, Gustot et al had shown that patients with infections in a background of cirrhosis have significantly higher incidence of septic shock and organ failure, require renal replacement therapy (RRT) more often and suffer decreased survival rates that compared to ICU patients without chronic liver diseases 12.

Around 18% of cirrhotic patients were admitted to our ICU due to hematemesis. Acute varicealhemorrhage (VH) is a medical emergency requiring intensive care management due to increased risk of rebleeding, infection and mortality. The immediate goal of therapy in these patients is to control bleeding, to prevent early recurrence (within 5 days) and prevent 6-week mortality, which is considered, by consensus, the main treatment outcome^17^. Renal impairment was seen in 18.5% of the cirrhotic patients in this study. According to Cholongitas et al, Renal failure (RF) is seen in 39% to 49% of the patients with cirrhosis admitted to the ICU 18. The most common causes of RF are hypovolemia (40%), bacterial infection (32%), parenchymal kidney disease (15%) and hepatorenal syndrome (HRS) (12%) 19.

We analyzed possible predictors of ICU-mortality in this cohort. SOFA score was the only independent predictor of mortality in the cirrhotic patients. This matches with other studies which showed that ICU prognostic scores have better predictability of outcome in the cirrhotic patients admitted to the ICU compared to conventional liver scores such as the Child-Pugh score and MELD score^20^, ^21^.

In the non-cirrhotic patients, ICU mortality was 53%. The most common presentations were complicated biliary obstruction, hematemesis, acute liver cell failure, and endoscopic complications. A minority of patients were admitted as a transitory stage before finding place in the specialized ICU.

Sixty-eight patients were admitted due to complications of biliary obstruction. Nineteen patients had liver cirrhosis in addition. Acute cholangitis is a known complication of biliary tract obstruction. Stasis to the biliary flow favors colonization and multiplication of bacteria. The associated increased intra-ductal pressure can lead to reflux of biliary contents and bacteremia that can eventually lead to septic shock and death 22.

The independent predictors of mortality in the non-cirrhotic patients were both SOFA score and the presence of sepsis.

## Conclusion

This study revealed that the mortality in cirrhotic patients admitted to the ICU is not higher than mortality in non-cirrhotic patients. Critically ill cirrhotic patients shouldn't be considered as poor ICU candidates and should be offered an ICU access. We can also conclude that SOFA score is a reliable prognostic indicator and could allow a better selection of patients who would most likely benefit from the ICU admission. In addition, earlier admission of critically ill cirrhotic patients before the development of multi-organ failure should be considered to improve patients' outcome.
